# Effect of red mud and citric acid on the mechanical properties and water resistance of magnesium oxychloride cement

**DOI:** 10.1038/s41598-026-55273-7

**Published:** 2026-05-27

**Authors:** Yanrong Wang, Yuanyuan Zhao, Runfang Zhou, Haitao Mao

**Affiliations:** 1 College of Urban and Rural Construction, Shanxi Agricultural University, Taigu, 030801 Shanxi China; 2 Hebei Vocational University of Technology and Engineering, Xingtai, 054000 Hebei China; 3 Shanxi Smart Water-Saving Technology Innovation Center, Taiyuan, 030000 Shanxi China

**Keywords:** Magnesium oxychloride cement, Citric acid, Water resistance, Mechanical properties, Engineering, Environmental sciences, Materials science

## Abstract

Despite its excellent mechanical and environmental properties, magnesium oxychloride cement (MOC) suffers from poor water resistance and high production costs, ultimately limiting its application. This study investigates the use of red mud (RM) and citric acid (C_6_H_8_O_7_) to develop a low-cost, water-resistant RM-based MOC (RM-MOC). The effects of varying RM/C_6_H_8_O_7_ contents on the setting time, compressive strength, water resistance, and microstructure of RM-MOC were analyzed. The addition of RM/C_6_H_8_O_7_ prolonged the setting time and enhanced water resistance; however, it reduced mechanical strength. Mechanistic analysis revealed that RM reduced 5Mg(OH)_2_·MgCl_2_·8H_2_O (Phase 5) formation in RM-MOC, whereas C_6_H_8_O_7_ facilitated the generation of M-S-H and Mg-Cl-Si-Al-H gels with homogeneous structures and high polymerization degrees. These gels refined the pore structure and densified RM-MOC, thereby improving water resistance and compensating for the strength loss due to reduced Phase 5 formation. Under the synergistic action of RM and C_6_H_8_O_7_, RM-MOC achieved a compressive strength of 59 MPa and softening coefficients of 0.8 (7 d) and 0.78 (28 d) even at a high RM proportion of 60% (RM/LBMP = 0.6), demonstrating effective waste valorization and simultaneous enhancement of material properties. This study provides a novel strategy for the development of high-performance, low-cost, and environmentally friendly magnesium cement materials.

**Supplementary Information:**

The online version contains supplementary material available at 10.1038/s41598-026-55273-7.

## Introduction

Red mud (RM) is the primary alkaline solid waste generated during alumina production^1,2^, with ~ 1.2–1.5 tons of RM being produced for every ton of alumina manufactured^3,4^. As one of the world’s largest alumina producers, China has accumulated > 100 million metric tons of RM. Owing to its high pH and heavy-metal content, RM poses serious risks to soil and water resources and adversely affects the ecological environment^5,6^. Moreover, large-scale RM stacking occupies vast land resources^7,8^ and incurs high maintenance costs, thereby increasing the cost of alumina production. Consequently, an effective solution is urgently required for the comprehensive recovery and utilization of RM.

Magnesium oxychloride cement (MOC) is an air-setting cementitious material that primarily comprises 5Mg(OH)_2_·MgCl_2_·8H_2_O (Phase 5) and forms via the reaction of light-burned magnesium powder (LBMP) with MgCl_2_ solution^9–11^. MOC is characterized by its early strength, wear resistance, thermal insulation, and fire resistance^12–14^. Consequently, this material has been incorporated into concrete, wall materials, fireproof boards, and decorative materials^15–17^. However, the widespread application of MOC is significantly limited by its inferior water resistance and higher production costs compared with those of ordinary Portland cement. Additionally, its main hydration products, Phases 3 and 5, convert to brucite upon contact with water, creating numerous pores and reducing strength^17,18^, significantly restricting the use of MOC in construction materials.

The type and dosage of mineral admixtures can markedly affect the mechanical properties and durability of cementitious materials through the modulation of hydration product composition and microstructural characteristics. In the slag/fly ash-based geopolymer-stabilized clay system, the synergistic regulation of the initial amount-of-substance ratios of CaO/Al_2_O_3_ and SiO_2_/Al_2_O_3_ plays a determining role in strength development and water stability^19^. Furthermore, the type of clay minerals (e.g., montmorillonite, kaolinite, illite) also exerts a notable influence on the strength and water stability of geopolymer-stabilized soils^20^. Studies have demonstrated that mineral admixtures can considerably reduce the production cost of MOC by altering its hydration process, hydration products, and microstructure, while also enhancing its water resistance to some extent^21,22^. For example, according to He et al^23^., pulverized fuel ash can improve the water resistance of MOC by promoting the formation of amorphous gels. Additionally, the incorporation of dredged sediment into MOC enhances the yield of the Mg-Cl-Si-Al-H gel, consequently enhancing both the water resistance and dimensional stability of the cement system^24^. Furthermore, metakaolin^25^, silica fume^26^, and incinerated sewage sludge ash^27^ have been shown to enhance the water resistance of MOC. Notably, RM contains large amounts of Fe_2_O_3_, Al_2_O_3_, SiO_2_, and CaO^28–30^, rendering it a promising mineral modifier. In this context, Liu et al^31^. reported that RM improved the mechanical performance and water resistance of magnesium phosphate cement by filling pores to create a denser structure and by promoting the formation of new gelatinous hydrates. Moreover, mixing ordinary Portland cement with RM produces RM-based cementitious materials through cement hydration and geopolymer reactions, yielding mechanical properties that surpass those of 32.5R cement^32^. It has also been reported that replacing 50% of the LBMP in MOC with RM enhances water resistance without significantly affecting strength^33^. The addition of 10%–20% RM also improves the durability and bonding performance of magnesium potassium phosphate cement^34^.

Although mineral admixtures can enhance the water resistance of MOC, their effectiveness often remains insufficient for practical applications. Consequently, studies have increasingly explored the use of chemical admixtures to enhance the water resistance of MOC. For instance, Chen et al^35^. found that phosphoric acid modifies the morphology of Phase 5 crystals in MOC, thereby improving its water resistance and increasing the softening coefficient to > 0.78. Other studies have indicated that the addition of sodium D-gluconate can chelate with Phase 5 in MOC, transforming it into a gel-like substance that enhances compactness and further improves water resistance^35^. Additionally, citric acid (C_6_H_8_O_7_) significantly enhances both the water resistance and strength of MOC^37,38^. Nevertheless, despite their effectiveness in enhancing water resistance, the relatively high cost of these acidic modifiers results in higher production costs for MOC.

To simultaneously enhance water resistance and reduce production costs, it is imperative to leverage the synergistic effects of acidic and mineral admixtures, while also evaluating their combined impact on mechanical performance. Such an integrated methodology represents a promising strategy for addressing the aforementioned challenges. Furthermore, as a bulk solid waste material with high utilization potential, RM is a widely recognized and government-endorsed recyclable resource. Its incorporation not only significantly reduces the cost of MOC but also contributes to waste management and carbon-emission reduction. Moreover, citric acid has been shown to enhance both water resistance and mechanical strength. Specifically, previous studies have explored the modification effects of RM and citric acid on MOC. However, the potential chemical synergy between the acidic modifiers and the highly alkaline RM has yet to be considered. For instance, ions such as Al^3+^ and Fe^3+^ in RM can form complexes with organic acids, which can retard hydration and alter the hydration products of the MOC system. It is therefore essential to clarify the interaction mechanism between RM and citric acid within the MOC system to promote the high-value utilization of industrial solid waste in MOC.

Thus, in the current study, RM and citric acid are employed as modifiers for MOC. Through experimental investigations, the synergistic effects of RM and citric acid on the setting time, compressive strength, and water resistance of RM-MOC are systematically evaluated. X-ray diffractometry (XRD), Fourier transform infrared (FTIR) spectroscopy, thermogravimetric–derivative thermogravimetry (TG-DTG), scanning electron microscopy (SEM), and energy-dispersive X-ray spectroscopy (EDS) are employed to examine the composition and microstructure of the MOC hydration products under the synergistic effect of RM and citric acid. This study aims to optimize the overall performance of RM-MOC while also promoting the utilization of RM waste, reduce environmental pollution, and lower the production cost of MOC to provide a new theoretical basis and technical pathway for the high-value recycling of RM and performance enhancement of MOC.

## Materials and methods

### Materials

RM-MOC was prepared using LBMP, magnesium chloride hexahydrate (MgCl_2_·6H_2_O), RM, and C_6_H_8_O_7_ in varying proportions. LBMP was produced by Yingkou Huiteng Refractory Co., Ltd. (Liaoning, China), and its active magnesium oxide (α-MgO) content was determined to be 60% using the hydration method. Bayer RM was purchased from Shandong Aluminum Co., Ltd. (Zibo, China). The RM was oven-dried at 105 °C for 24 h, ground in a ball mill for ~ 40 min and sieved to obtain a particle size of < 200 mesh. MgCl_2_·6H_2_O was procured from Shanghai Minhang Shenlong Light Chemical Co., Ltd. (Shanghai, China) and citric acid was purchased from Tianjin Beichen Fangzheng Reagent Co., Ltd. (Tianjin, China).

**Fig. 1:**
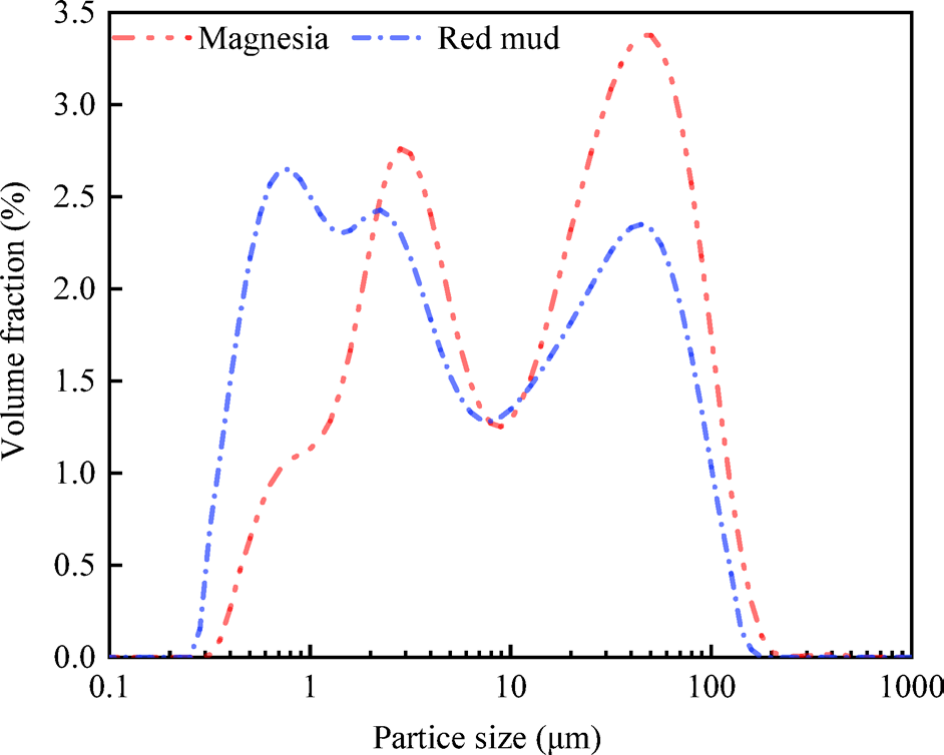
Particle size distribution of magnesia and RM.

**Fig. 2:**
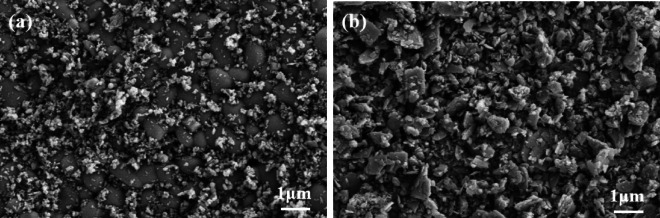
SEM images of raw materials: (**a**) LBMP and (**b**) RM.

Analysis of the particle size distribution (Fig. 1) reveal that RM possesses smaller particle sizes with a more uniform dispersion than LBMP. SEM images of LBMP and RM reveal that the LBMP particles are predominantly spherical (Fig. 2a), whereas the RM particles appear as small flakes and irregular polygons (Fig. 2b). Additionally, RM exhibits a finer particle size and more compact structure than LBMP.

**Fig. 3:**
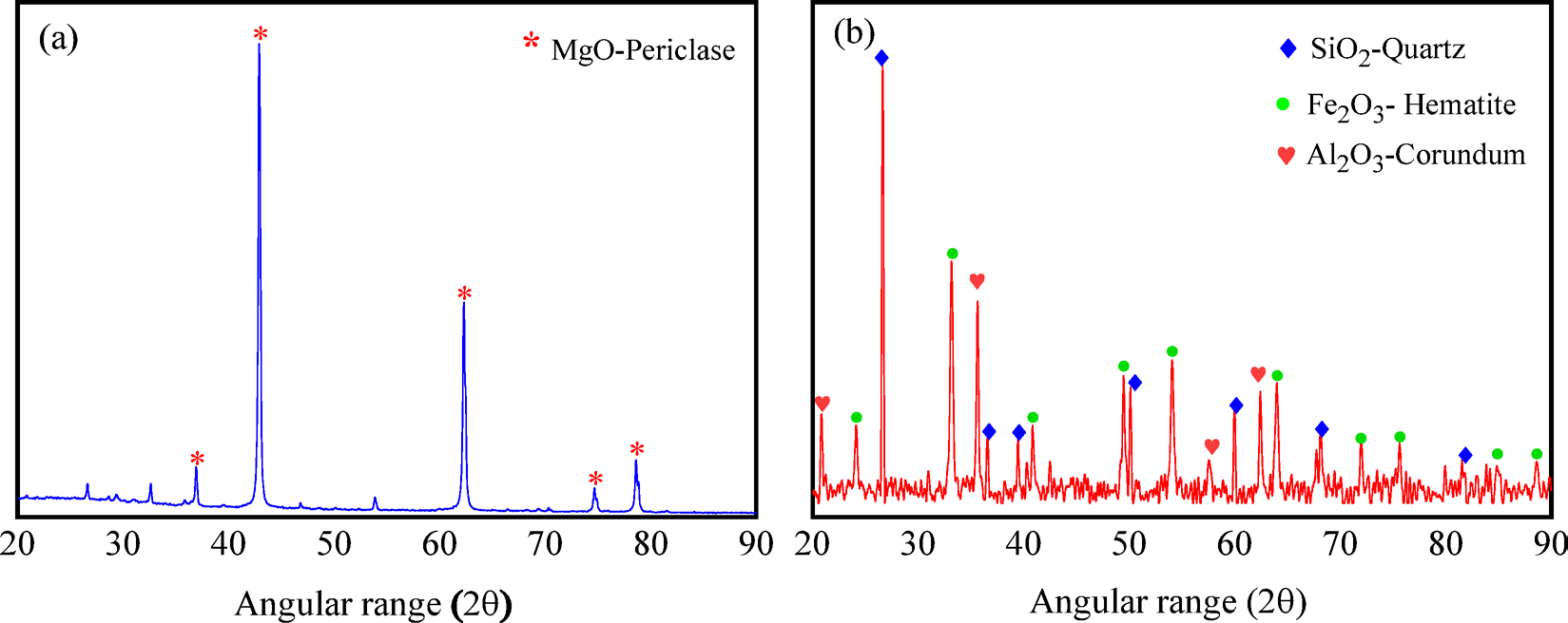
XRD patterns of raw materials: (**a**) LBMP and (**b**) RM.

The XRD patterns of LBMP and RM indicate that the main phase of LBMP is MgO, while SiO_2_, Fe_2_O_3_, and Al_2_O_3_ are the principal components of RM (Fig. 3). The X-ray fluorescence (XRF) results show that SiO_2_, Fe_2_O_3_, and Al_2_O_3_ account for 81.9% of the total oxide content in RM, whereas the MgO content in LBMP is 93.8% (Table 1).

**Table 1:** Oxide composition of raw materials.

Raw material	Mass fraction of the samples (%)												
	MgO	Al_2_O_3_	SiO_2_	*P*_2_O_5_	SO_3_	K_2_O	CaO	Na_2_O	Fe_2_O_3_	TiO_2_	V_2_O_5_	MnO	ZrO_2_
LBMP	93.8	0.36	3.99	0.03	0.09	0.02	1.47	0.02	0.29				
RM	0.13	22.6	14.6	0.22	0.69	0.18	0.87	10.1	44.7	5.37	0.197	0.094	0.084

### Experimental procedures

#### Sample formulation

Based on the study by Li et al.^39^ and preliminary experiments, the amount-of-substance ratio of n(α-MgO)/n(MgCl_2_·6H_2_O)/n(H_2_O) ratio was set to 7:1:16, while the RM/LBMP mass ratio was set to 0, 0.1, 0.2, 0.3, 0.4, 0.5, and 0.6. Considering a previous report that 1% C_6_H_8_O_7_ addition can effectively improve the fluidity and volume stability of MOC^40^, dosages of 0.5%, 1%, and 1.5% (by weight of LBMP) were investigated. Table 2 lists the compositions of the prepared samples. For clarity, the samples were labeled as RxCy, where R and C represent the amounts of RM and C_6_H_8_O_7_ added, respectively. In this study, the water-to-solid ratio (W/S) was fixed at 0.27.

**Table 2:** Mix proportions of RM-MOC samples (g).

Sample	LBMP	RM	MgCl_2_·6H_2_O	H_2_O	C_6_H_8_O_7_	W/S
R0C0	1000	0	435	385.71	0	0.27
R1C0	1000	100	435	412.71		
R2C0	1000	200	435	439.71		
R3C0	1000	300	435	466.71		
R4C0	1000	400	435	493.71		
R5C0	1000	500	435	520.71		
R6C0	1000	600	435	547.71		
R0C1	1000	0	435	385.71	10	
R1C1	1000	100	435	412.71		
R2C1	1000	200	435	439.71		
R3C1	1000	300	435	466.71		
R4C1	1000	400	435	493.71		
R5C1	1000	500	435	520.71		
R6C1	1000	600	435	547.71		
R2C0.5	1000	200	435	439.71	5	
R4C0.5	1000	400	435	493.71		
R6C0.5	1000	600	435	547.71		
R2C1.5	1000	200	435	439.71	15	
R4C1.5	1000	400	435	493.71		
R6C1.5	1000	600	435	547.71		

#### Sample preparation

**Fig. 4:**
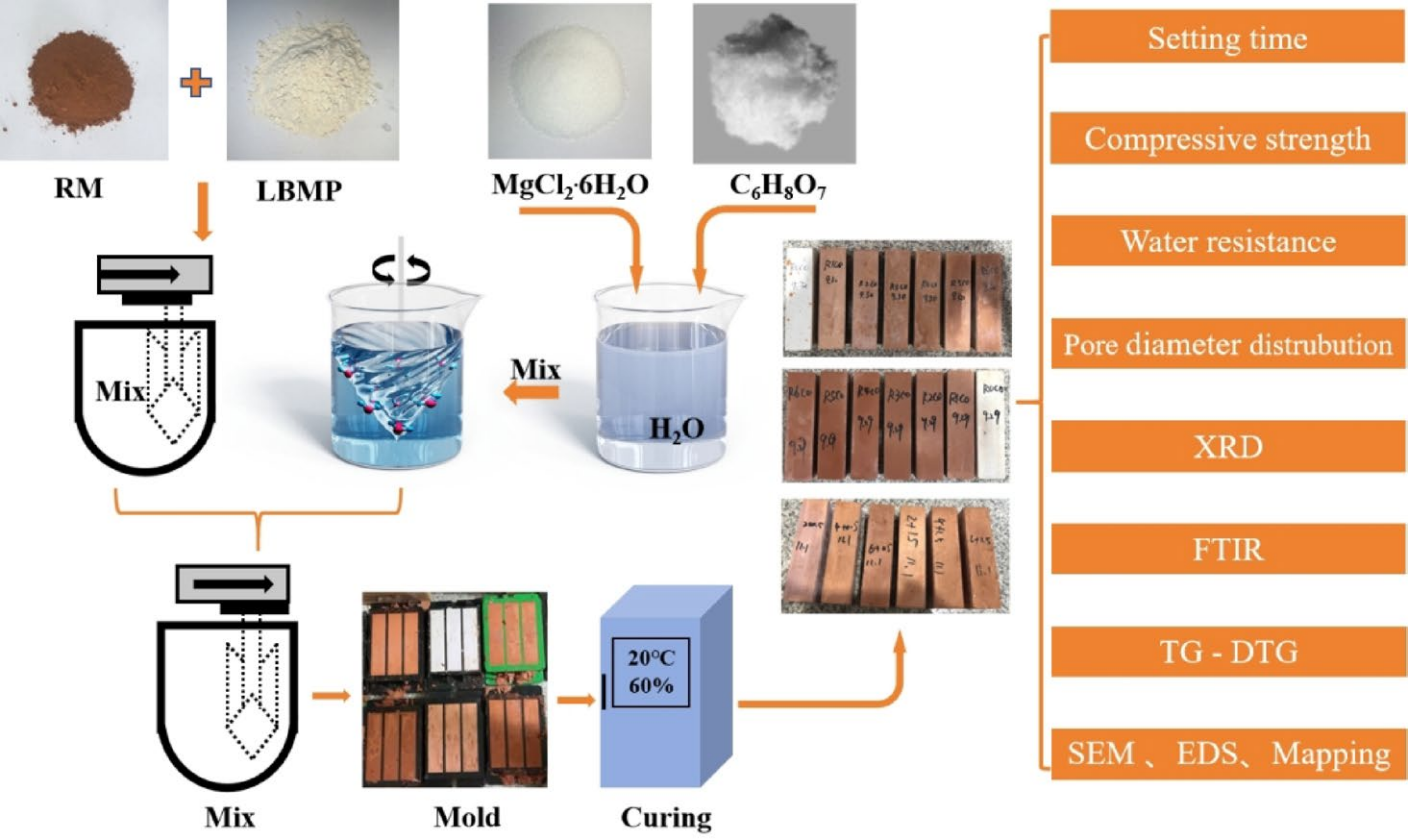
Preparation and characterization procedures of MOC composites. (The figure was created by WPS Office (version 12.1.0.25225, https://www.wps.cn/)).

The samples were prepared (Fig. 4) in a planetary cement sand mixer (NJ-160, Maifang Instruments and Equipment Co., Ltd., Wuxi, China) according to the following procedure. First, RM and LBMP were dry-mixed, followed by the addition of MgCl_2_·6H_2_O and C_6_H_8_O_7_ in H_2_O, and subsequent mixing (60 s low speed → 90 s rest → 60 s high speed). The obtained samples were then cast into 40 mm × 40 mm × 160 mm polyvinyl chloride molds, vibrated for 60 s, and cured for 24 h under ambient conditions before demolding. Finally, the samples were cured at 20 ± 3 °C and 60 ± 5% relative humidity for 3,7, 28, and 42 d.

### Test methods

The setting times of the RM-MOC paste were measured using the Vicat apparatus method in accordance with the National Standard of China GB/T 1346–2024. The initial setting time was measured from the moment water was added, with periodic assessments performed using the initial needle until the penetration depth reached a distance of 4 *±* 1 mm from the baseplate. The mold was then inverted, and the final setting time was assessed using the final-setting needle. The final set was characterized as the point at which needle penetration did not exceed 0.5 mm and the annular attachment no longer left a visible mark on the surface.

The compressive strength of the MOC was tested according to the ISO method of the China National Standard GB/T17671-2021. Compressive tests were conducted using a universal testing machine (WE-300B, Jinan Zhongluchang Testing Machine Manufacturing Co., Ltd., Jinan, China) at a loading rate of 2400 N/s. The compressive strength was determined as the average value of six test blocks and calculated as follows:

1 $$R_c=\frac{F_c}{A}$$

where *R*_*c*_ is the compressive strength (MPa), *F*_*c*_ denotes the ultimate load (N), and *A* is the compressive area (mm^2^).

The water resistance of RM-MOC was systematically evaluated by initially curing the specimen in air for 28 d followed by subsequent immersion in water for 7 and 28 d. The samples were then removed from the water, surface-dried, and tested for residual compressive strength. The softening coefficient (*R*_*w*_) was calculated using the following formula:

2 $$R_w=\frac{C_n}{C_{28}}$$

where *C*_*n*_ is the residual compressive strength of the test block after curing in air for 28 d and soaking in water for *n* d, and *C*_*28*_ (MPa) is the compressive strength of the MOC after 28 d of curing.

After strength testing, the broken pieces were soaked in anhydrous alcohol for 24 h then dried in an oven at 40 °C for a further 24 h to halt the hydration process. The pore distributions of the samples were determined using the Brunauer–Emmett–Teller (BET) nitrogen adsorption technique (Quadrasorb, Anton Paar). The sample pieces were pulverized into a fine powder, and the hydration phases were examined using XRD (XD8 Advance, Bruker, Karlsruhe, Germany) with Cu Kα radiation at 60 kV and 80 mA and a scan rate of 0.02°/s. FTIR spectroscopy was performed from 400 to 4000 cm^− 1^ at 1 cm^− 1^ resolution. TG-DTG (TGA 5500) analysis was performed on pre-processed flakes heated at 10 °C/min under a N_2_ atmosphere (30–800 °C). The microstructural morphologies were revealed by SEM (Gemini 300, ZEISS, Oberkochen, Germany), and the elemental compositions were determined using EDS (SU8100, Hitachi, Tokyo, Japan).

## Results

### Setting time

**Fig. 5:**
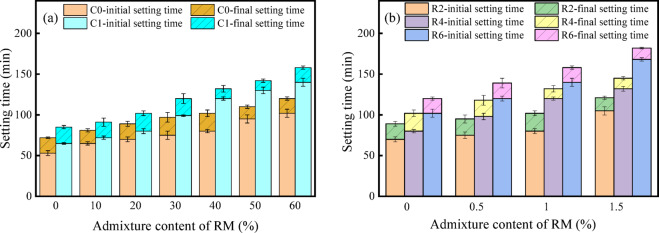
Setting times of MOC samples.

Figure 5a shows the effects of different RM/C_6_H_8_O_7_ ratios on the setting time of the MOC slurry. Without the addition of C_6_H_8_O_7_, the setting time increased with RM content (Fig. 5a). Specifically, the addition of 60% RM gave initial and final setting times of 102 and 120 min, respectively, representing increases of 49 and 48 min compared with the RM-free systems. The addition of 1% C_6_H_8_O_7_ to the RM-MOC slurry resulted in a longer setting time compared with those of the systems containing RM alone. For instance, at 60% RM content, the initial and final setting times reached 140 and 158 min, respectively, representing an extension of 38 min compared with the corresponding times in the absence of C_6_H_8_O_7_. Maintaining a constant RM dosage (Fig. 5b), the setting time increased with increasing C_6_H_8_O_7_ dosage, giving optimal initial and final setting times of 168 and 182 min, respectively, for the system containing 1.5% C_6_H_8_O_7_ and 60% RM. These results indicate that the RM-MOC setting time increases significantly with increasing RM and C_6_H_8_O_7_ contents. When used simultaneously, RM and C_6_H_8_O_7_ exhibit a synergistic retardation effect, improving workability and facilitating heat dissipation during cement hydration.

### Compressive strength

**Fig. 6:**
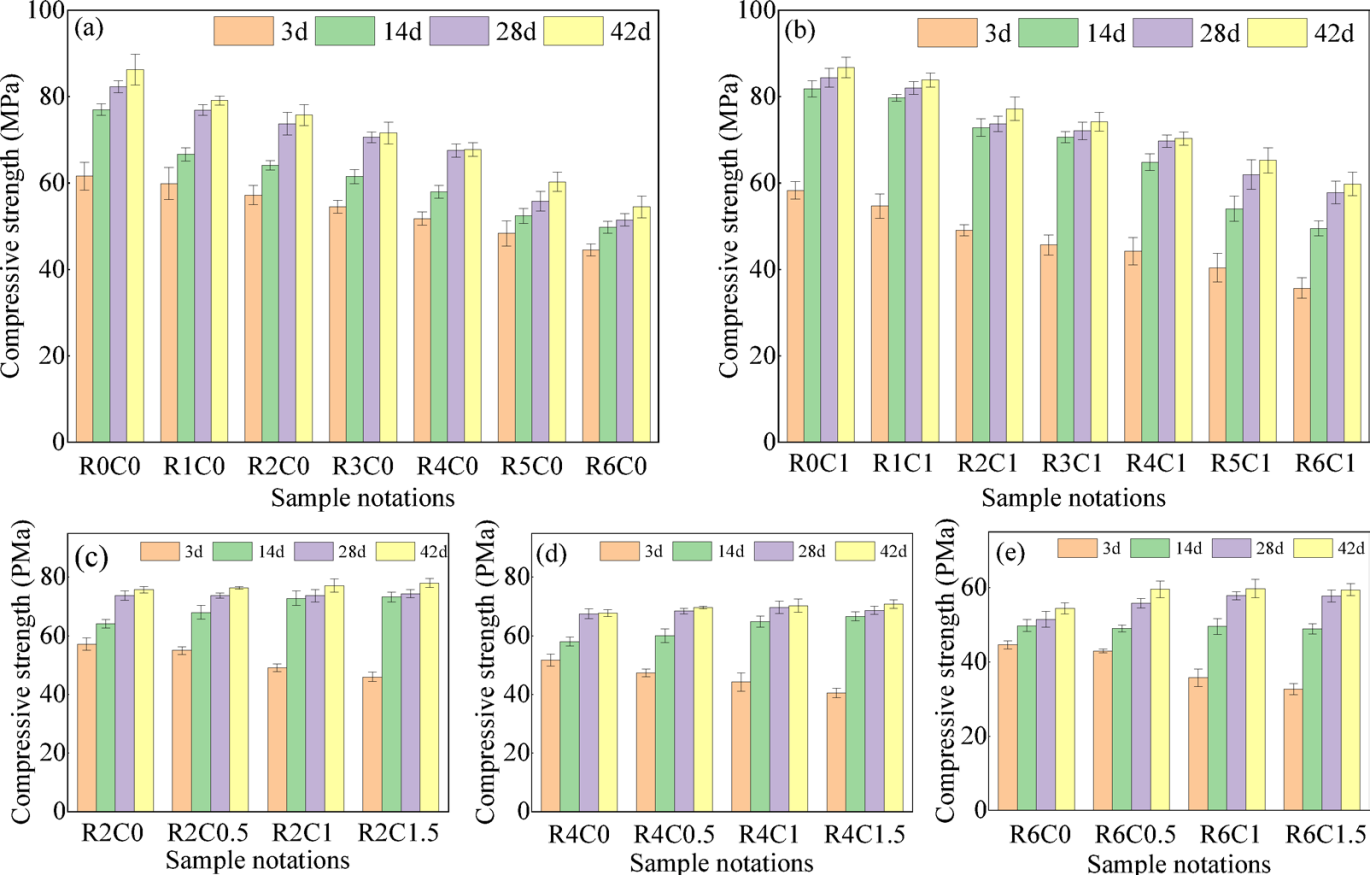
Compressive strengths of MOC samples. (The figure was created by Origin(Version: OriginPro 2021 9.8.0.200, https://www.originlab.com/)).

Figure 6 illustrates the synergistic effects of RM/C_6_H_8_O_7_ on the compressive strength of MOC after curing for 3, 14, 28, and 42 d. In the absence of C_6_H_8_O_7_, the compressive strength exhibited a progressive reduction from 86.23 MPa (R0C0) to 54.48 MPa (R6C0) after 42 d of curing, representing a reduction of 36.8% with increasing RM incorporation (Fig. 6a). Upon the addition of 1% C_6_H_8_O_7_ (Fig. 6b), a similar decreasing trend was observed, wherein the compressive strength declined from 86.77 MPa (R0C1) to 59.77 MPa (R6C1) after 42 d, corresponding to 31.1% reduction.

These results indicate that the incorporation of RM reduced the compressive strength of RM-MOC, while the addition of 1% C_6_H_8_O_7_ lowered the extent of mechanical performance degradation from 36.8% to 31.1%. These findings demonstrate that the incorporation of C_6_H_8_O_7_ significantly alleviates the decline in mechanical properties induced by an elevated RM content, highlighting a synergistic enhancement effect between RM and C_6_H_8_O_7_ in improving the mechanical performance of RM-MOC.

To further explore the synergistic effects of C_6_H_8_O_7_ dosage on the mechanical properties of RM-MOC, the compressive strength was systematically evaluated at fixed RM incorporation levels of 20%, 40%, and 60%. After 3 d of curing, the RM-MOC strength exhibited a consistent decreasing trend with higher C_6_H_8_O_7_ content (Fig. 6c and e). Specifically, for 20% RM incorporation, the strength declined from 57.2 MPa (R2C0) to 46.0 MPa (R2C1.5), corresponding to a reduction of 19.58%. In the case of 40% RM addition, the compressive strength decreased from 51.79 MPa (R4C0) to 40.56 MPa (R4C1.5), reflecting a 21.68% reduction. Similarly, at 60% RM incorporation, the strength dropped from 45.55 MPa (R6C0) to 32.71 MPa (R6C1.5), representing a more substantial reduction of 28.19%. However, after curing for 14, 28, and 42 d, the compressive strength showed limited yet consistent improvements with increasing C_6_H_8_O_7_ content across all RM levels. After 42 d, increasing the C_6_H_8_O_7_ dosage resulted in strength gains of 3.0%, 4.5%, and 9.1% for RM incorporation levels of 20%, 40%, and 60%, respectively.

Overall, these results reveal that C_6_H_8_O_7_ exhibits an inhibitory effect on the early-age strength development of RM-MOC. As the curing age increases, the strength loss induced by high levels of RM incorporation is effectively compensated, and long-term strength development is enhanced. Both the inhibitory and enhancing effects become more pronounced at higher RM incorporation levels, further demonstrating the synergistic reinforcement between RM and C_6_H_8_O_7_ in improving the mechanical performance of RM-MOC. Notably, under this synergistic action, a high compressive strength of 59 MPa (R6C1/R6C1.5) was maintained even with 60% RM incorporation, which meets the strength requirement of P.O 52.5 ordinary Portland cement.

### Water resistance

**Fig. 7:**
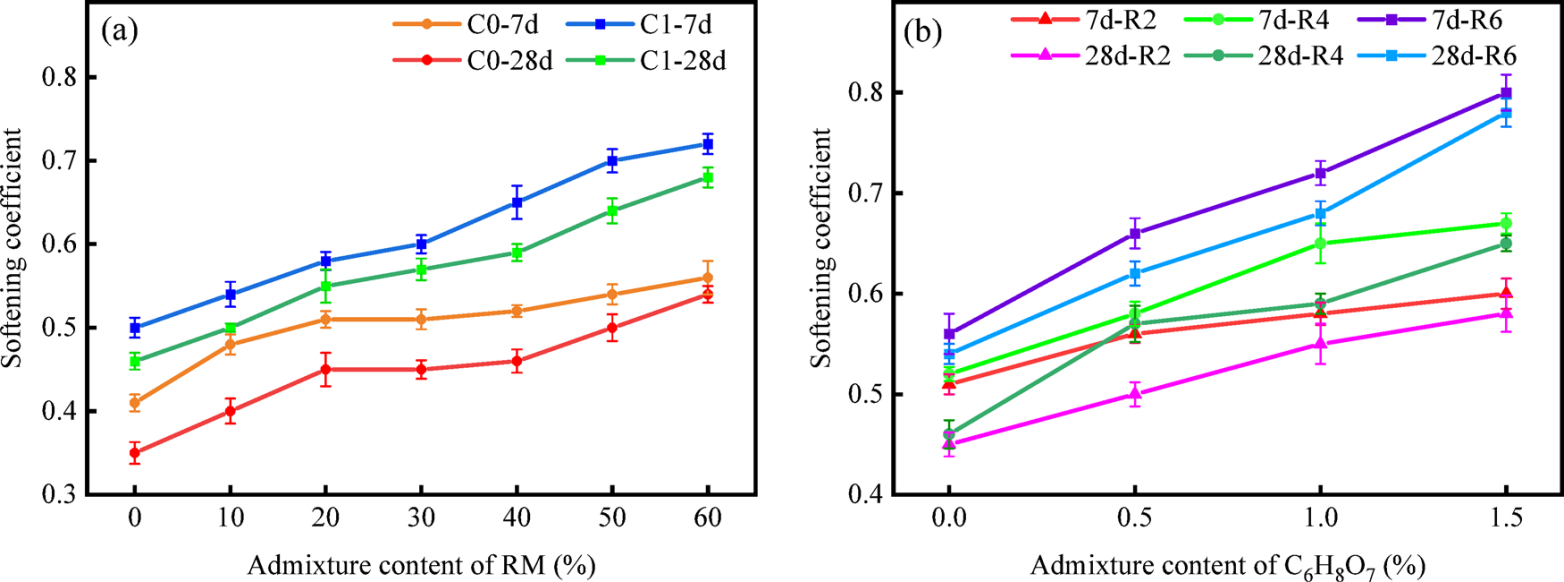
Softening coefficients of MOC samples after immersion in water.

Figure 7 illustrates the synergistic influence of RM and C_6_H_8_O_7_ on the softening coefficient of RM-MOC. After 7 and 28 d of immersion, the softening coefficient increased consistently with higher RM content (Fig. 7a), and the addition of 1% C_6_H_8_O_7_ further enhanced the softening coefficient across all RM incorporation levels. For instance, R6C0 exhibited softening coefficients of 0.56 and 0.50 after 7 and 28 d of immersion, respectively, corresponding to increases of 42.7% and 46.5% relative to the R0C0 group (0.41 and 0.35). This result confirms that RM incorporation significantly enhances water resistance. Furthermore, with the addition of 1% C_6_H_8_O_7_, the R6C1 group achieved values of 0.72 and 0.68, reflecting improvements of 25% and 32% compared with R6C0 (0.56 and 0.54), and 44% and 47.8% compared with R0C1 (0.50 and 0.46). These results demonstrate that both RM and C_6_H_8_O_7_ independently enhance the water resistance of RM-MOC. Moreover, their combined use, particularly at high RM content, substantially improves the long-term water resistance, highlighting a synergistic enhancement mechanism between RM and C_6_H_8_O_7_ in optimizing the durability of RM-MOC.

To further explore the synergistic effects of C_6_H_8_O_7_ dosage on the softening coefficient of RM-MOC, this parameter was systematically evaluated at fixed RM incorporation levels of 20%, 40%, and 60%. As shown in Fig. 7b, the softening coefficient of RM-MOC increased with higher C_6_H_8_O_7_ content. Specifically, for 20% RM incorporation, the softening coefficients after 7 and 28 d of immersion increased from 0.51 to 0.45 (R2C0) to 0.60 and 0.58 (R2C1.5), representing improvements of 17.65% and 28.89%, respectively. For samples containing 40% RM, the values rose from 0.52 to 0.46 (R4C0) to 0.67 and 0.65 (R4C1.5), reflecting enhancements of 28.85% and 41.3%. Notably, at 60% RM incorporation, the softening coefficients exhibited a substantial increase from 0.56 to 0.54 (R6C0) to 0.80 and 0.78 (R6C1.5), demonstrating significant gains of 43% and 44%, respectively. These findings indicate that the greatest improvement in the water resistance of RM-MOC due to C_6_H_8_O_7_ addition was achieved at 60% RM incorporation. Furthermore, the combination of 60% RM and 1.5% C_6_H_8_O_7_ optimized the synergistic effect, notably enhancing the water resistance of this material.

### Microstructural characterization

As detailed above, the synergistic effect of 1.5% C_6_H_8_O_7_ and 60% RM significantly enhanced the setting time, mechanical properties, and water resistance of MOC. The mechanisms underlying these improvements were elucidated by subjecting the R6C1.5 formulation to detailed microstructural analysis.

#### Pore diameter distribution

**Fig. 8:**
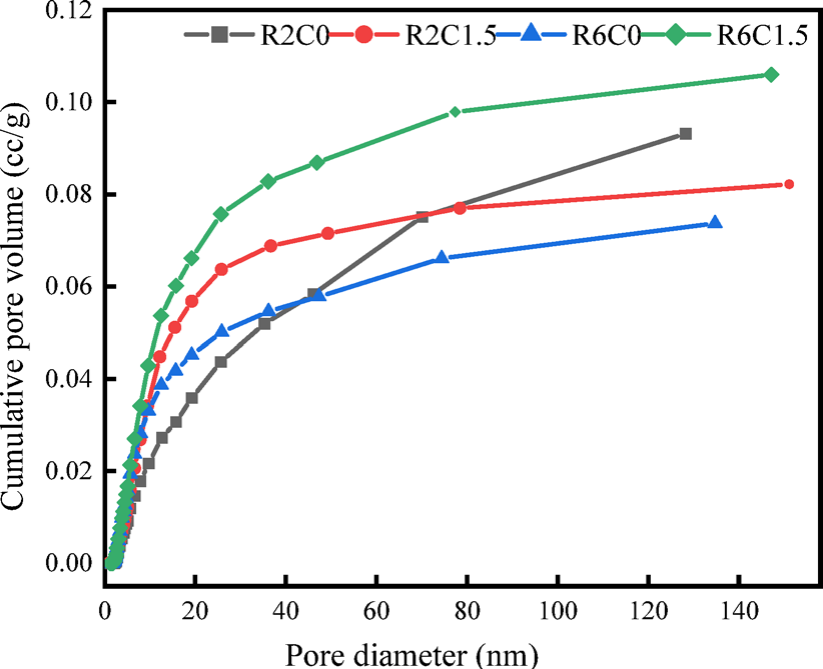
Pore diameter distributions of RM-MOC samples after curing for 28 d.

Figure 8; Table 3 illustrate the synergistic effects of different RM and C_6_H_8_O_7_ incorporation levels on the pore size distribution and pore structure characteristics of RM-MOC cured for 28 d. The total pore volume increased in the order R6C1.5 > R2C0 > R2C1.5 > R6C0. The cumulative pore volumes of R6C1.5 and R2C1.5 increased rapidly at pore sizes ≤ 20 nm and then more gradually above 20 nm. These results indicate a higher proportion of sub-20-nm pores in the R6C1.5 and R2C1.5 samples.

**Table 3:** Pore structure characteristics of RM-MOC samples after curing for 28 d.

Sample notations	Surface area (m^2^)	Average pore diameter (nm)	Proportion of different pore sizes/%		
			≤ 20 nm	20–100 nm	> 100 nm
R2C0	24.913	7.366	38.62%	42.13%	19.50%
R2C1.5	34.145	6.606	64.25%	29.69%	6.29%
R6C0	33.058	3.371	61.02%	28.34%	10.30%
R6C1.5	42.777	3.505	69.38%	24.94%	5.38%

Wu et al^41^. previously classified pore sizes as harmless (< 20 nm), less harmful (20–100 nm), harmful (100–200 nm), and multi-harmful (> 200 nm). Upon the incorporation of 60% RM to RM-MOC, the specific surface areas of R6C0 and R6C1.5 increased compared with those of R2C0 and R2C1.5; however, the average pore volume decreased, indicating that R6C0 and R6C1.5 contained more small pores. Additionally, compared with R2C0, the porosity of R6C0 decreased: the proportion of harmless pores increased from 38.62% to 61.02%, whereas that of harmful pores decreased from 19.50% to 10.30%. Furthermore, R6C1.5 contained more harmless pores (69.38%) than R2C1.5 (64.25%), while exhibiting fewer harmful pores. This was primarily attributed to the physical filling effect of the fine RM particles. Compared with R2C0 and R6C0, the incorporation of 1.5% C_6_H_8_O_7_ into RM-MOC (R2C1.5 and R6C1.5) increased the specific surface area and decreased the average pore size. Specifically, the proportion of harmless pores in R2C1.5 increased from 38.62% to 64.25%, whereas that of harmful pores decreased from 19.50% to 6.29%. Moreover, the proportion of harmless pores of R6C1.5 increased from 61.02% to 69.38%, whereas the proportion of harmful pores decreased from 10.3% to 5.38%. This indicates that C_6_H_8_O_7_ significantly increased the proportion of harmless pores while reducing that of harmful pores, thereby densifying the microstructure.

#### Characteristics of the hydration products

##### XRD analysis

**Fig. 9:**
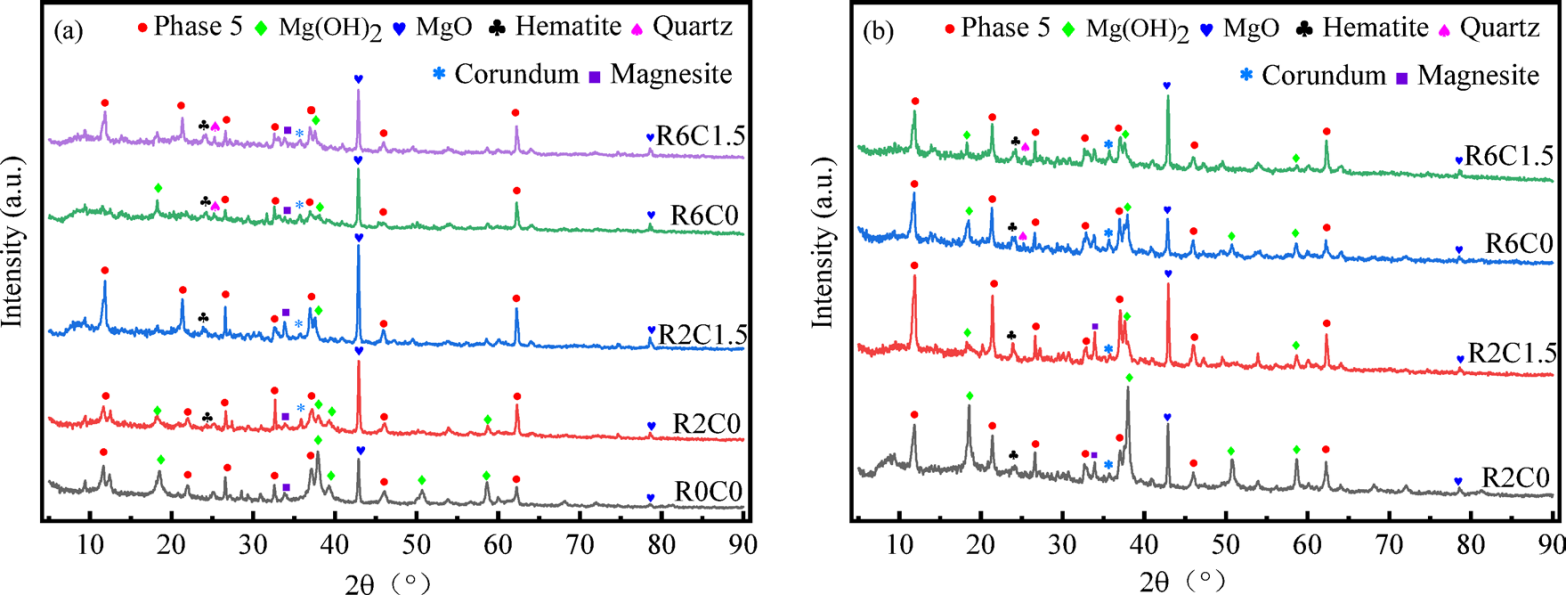
XRD patterns of MOC samples (**a**) after curing for 28 d and (**b**) after immersion in water for 28 d.

Figure 9 shows the XRD patterns of the RM-MOC samples after curing for 28 d and soaking in water for 28 d. As shown in Fig. 9a, the samples cured for 28 d exhibit pronounced Phase 5 diffraction peaks, indicating that Phase 5 is the main crystalline phase. Upon increasing the RM content, the diffraction peaks corresponding to the Phase 5 and Mg(OH)_2_ components of R0C0, R2C0, and R6C0 gradually weakened. This phenomenon is likely due to the physical barrier effect of RM, which inhibits the formation of Phase 5, while certain active components within RM simultaneously consume Mg(OH)_2_. Compared with R2C0 and R6C0, R2C1.5 and R6C1.5 exhibit more pronounced Phase 5 diffraction peaks and weaker Mg(OH)_2_ diffraction peaks. The retarding effect of C_6_H_8_O_7_ creates favorable conditions for the reaction between SiO_2_, Al_2_O_3_, and other components in RM with MgO, thereby promoting the consumption of Mg(OH)_2_ and suppressing its formation^42^. Moreover, a small number of diffraction peaks corresponding to SiO_2_, Fe_2_O_3_, and Al_2_O_3_ can be observed in the sample with RM addition, consistent with the composition of RM^43^. Moreover, MgCO_3_ was formed by the reaction between Mg(OH)_2_ and bicarbonate phases in the samples.

Figure 9b presents the XRD patterns of the RM-MOC samples after immersion in water for 28 d, revealing that the Phase 5 diffraction peaks for R2C0 and R6C0 exhibit similar intensities, whereas the Mg(OH)_2_ diffraction peak is more intense for R2C0 than for R6C0, suggesting a higher Mg(OH)_2_ content in R2C0. Additionally, the Phase 5 diffraction peak for R2C1.5 is stronger than that for R2C0, and the Mg(OH)_2_ diffraction peak becomes weaker, almost disappearing. Similar Phase 5 peaks were observed for R6C1.5 and R6C0, while the Mg(OH)_2_ diffraction peak for R6C1.5 was slightly weaker than that for R6C0, indicating that the addition of an appropriate amount of RM/C_6_H_8_O_7_ can inhibit Phase 5 hydrolysis and enhance water resistance.

##### FTIR analysis

**Fig. 10:**
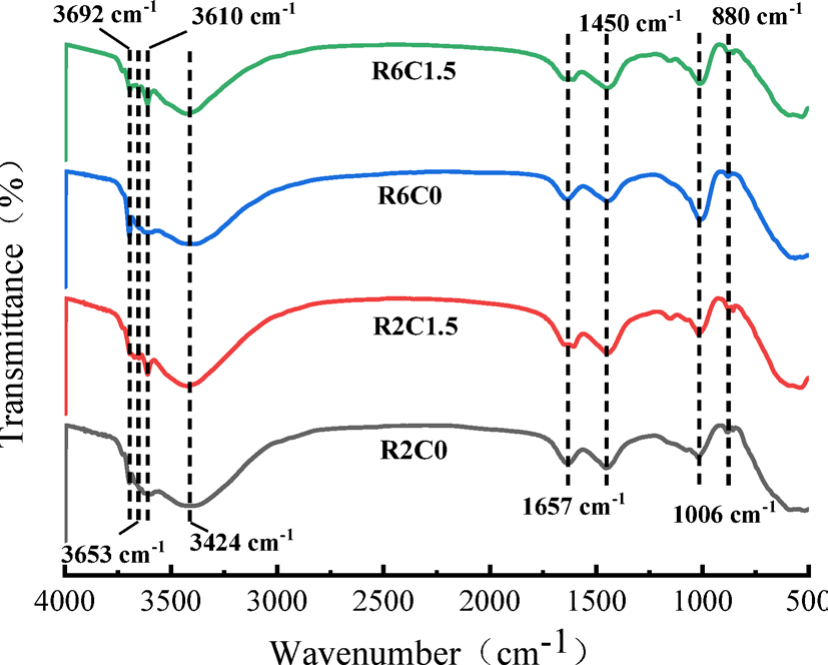
FTIR spectra of different RM-MOC samples after curing for 28 d.

Figure 10 shows the FTIR spectra of the RM-MOC samples containing different RM/C_6_H_8_O_7_ dosages after curing for 28 d. The O–H stretching vibrations of Phase 5 and Mg(OH)_2_ produced absorption peaks at 3610 and 3692 cm^− 1^, respectively^44^. After the addition of C_6_H_8_O_7_, the peak at 3610 cm^− 1^ became more pronounced for R2C1.5 and R6C1.5 than for R2C0 and R6C0, whereas the peak at 3692 cm^− 1^ decreased in intensity. This indicates that R2C1.5 and R6C1.5 contain a higher proportion of Phase 5 and a lower Mg(OH)_2_ content, consistent with the XRD results. Additionally, the absorption peaks at 3424 and 3453 cm^− 1^ can be attributed to the O–H stretching vibrations of hydrogen-bonded water in Phase 5^45^. Furthermore, the absorption peak at 1657 cm^− 1^ may be ascribed to the changes in MgCl_2_∙6H_2_O caused by the O–H bending vibration in H_2_O^14^, while the peak at 1450 cm^− 1^ corresponds to the characteristic stretching vibration of C = O in the carbonates^46^. Moreover, the observation of Si–O–Si and Si–O stretching vibrations at 1006 cm^–1 47^ indicate the presence of silicate or quartz in RM-MOC, originating from the RM raw material.

##### Thermal stability analysis

**Fig. 11:**
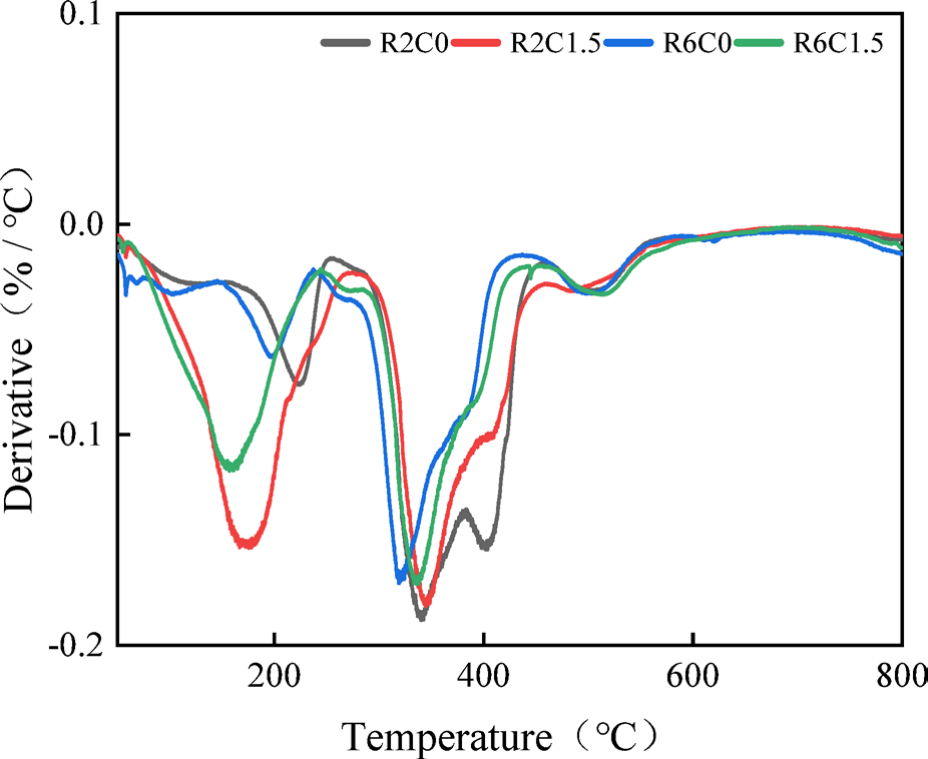
DTG curves of MOC samples after curing for 28 d.

**Fig. 12:**
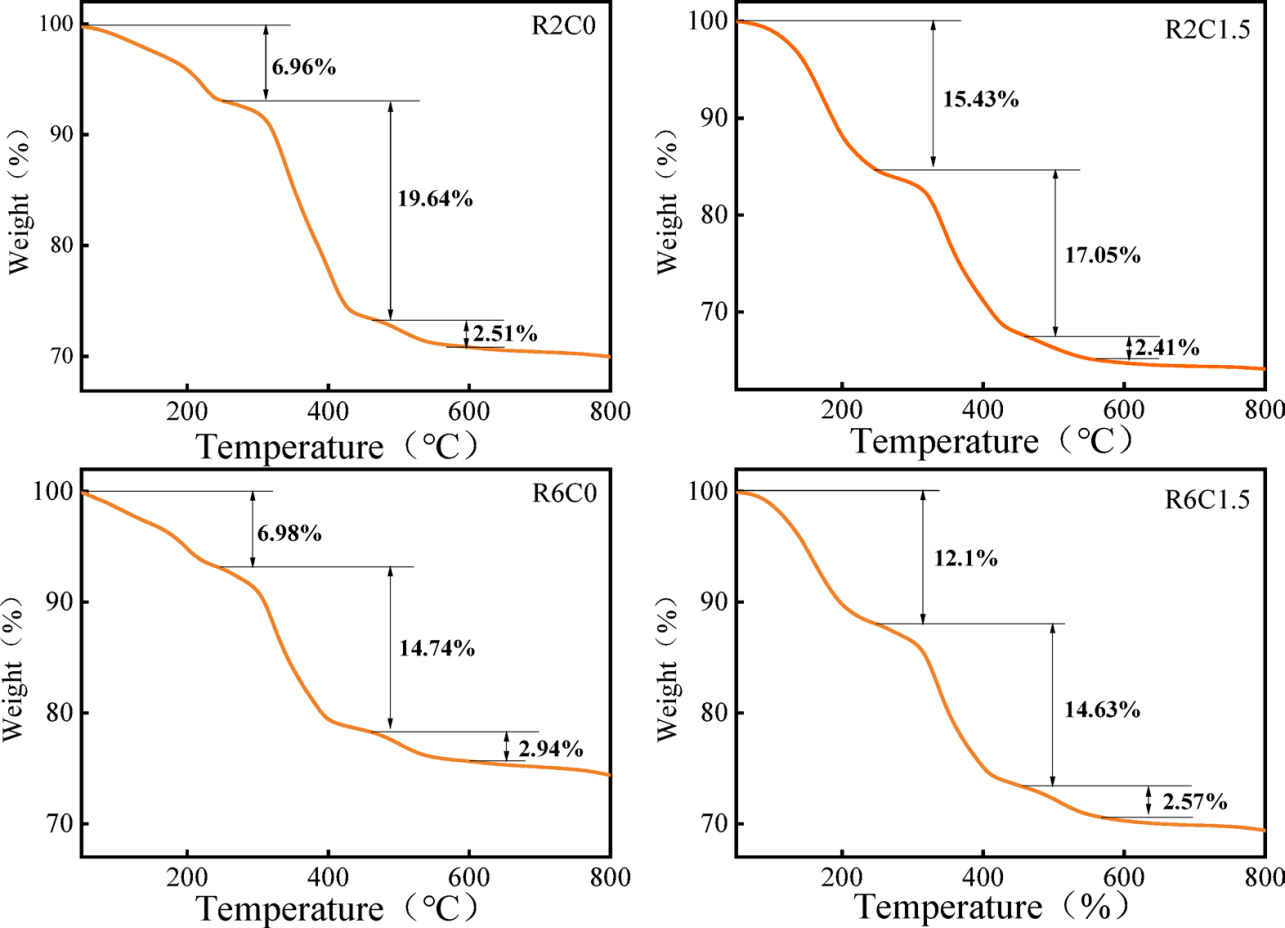
TG curves of MOC samples after curing for 28 d.

Figures 11 and 12 present the TG-DTG profiles of the RM-MOC samples containing various RM/C_6_H_8_O_7_ dosages after curing for 28 d. The DTG curves exhibit three distinct decomposition stages. Specifically, in Stage Ⅰ (40–250 °C), mass loss mainly results from the removal of free and partially bound water, causing the Phase 5 structure to loosen. In Stage II (250–460 °C), Phase 5 decomposes into Mg(OH)_2_ and MgCl_2_. Subsequently, MgCl_2_ partially hydrolyzes to HCl, and Mg(OH)_2_ decomposes into MgO and H_2_O. When the temperature exceeds 500 °C, MgCO_3_ decomposes with the release of CO_2_.

As shown in Fig. 12, while R2C0 and R6C0 exhibit comparable mass losses during the first heating stage (0–250 °C), R2C0 shows a significantly greater mass loss (19.64%) than R6C0 (14.74%) in the 250–460 °C range, indicating a higher Mg(OH)_2_ content in R2C0. Additionally, R6C0 and R6C1.5 exhibit similar mass loss magnitudes in Stage II; however, the mass loss of R6C1.5 in Stage Ⅰ (12.1%) exceeds that of R6C0 (6.98%), suggesting the formation of a new hydrated gelling phase or citric acid complex in R6C1.5.

#### SEM analysis

**Fig. 13:**
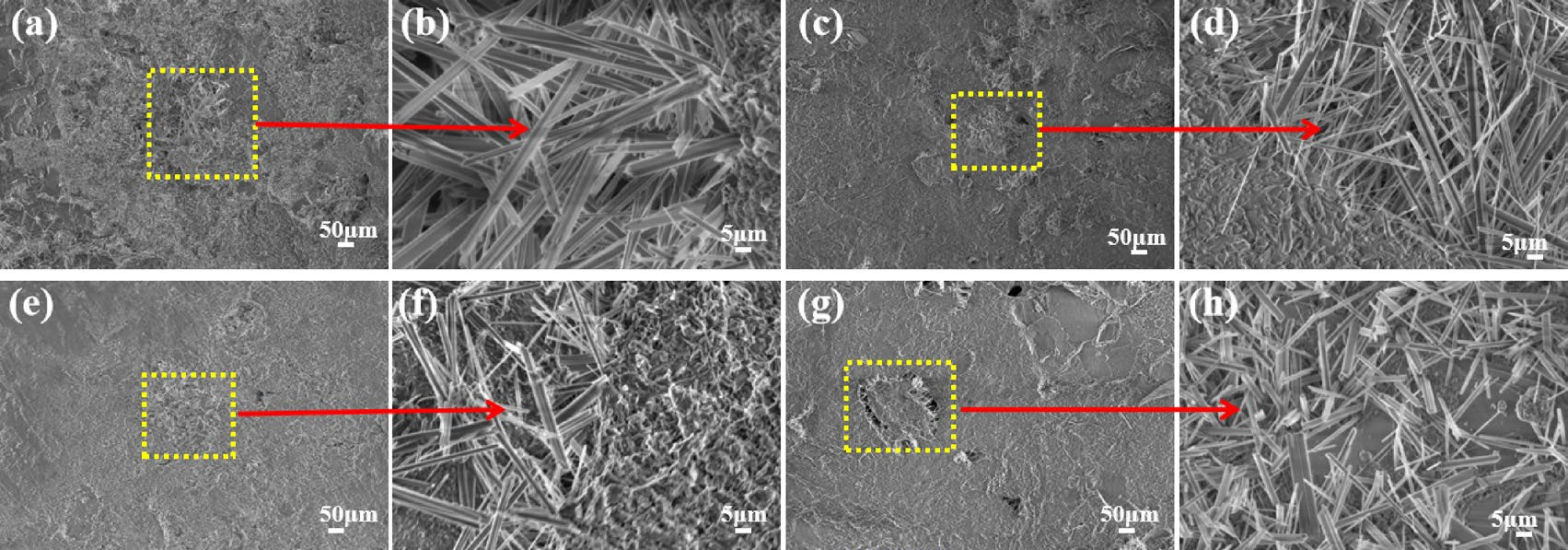
SEM images of RM-MOC samples at different magnifications after curing for 28 d: (**a**) R2C0 at 50 μm; (**b**) R2C0 at 5 μm; (**c**) R2C1.5 at 50 μm; (**d**) R2C1.5 at 5 μm; (**e**) R6C0 at 50 μm; (**f**) R6C0 at 5 μm; (**g**) R6C1.5 at 50 μm; and (**h**) R6C1.5 at 5 μm.

Figure 13 shows the SEM images of the RM-MOC samples with varying RM and C_6_H_8_O_7_ contents after curing for 28 d. All sample pores are clearly filled with needle-and-rod crystals, characteristic of Phase 5, which form internal pore surfaces that generate compact microstructures, thereby enhancing the mechanical strength^28^. Numerous acicular Phase 5 whiskers can be observed in R2C0 and R2C1.5, whose surfaces are covered with interwoven and flocculent networks. Compared with R2C0 and R2C1.5, R6C0 and R6C1.5 exhibit smaller pores, and their surfaces are finer and denser. Additionally, R6C0 and R6C1.5 incorporate distinct needle-like crystals and several gel-like substances. This phenomenon can be attributed to elevated RM incorporation, which reduces the concentration of α-MgO in the RM-MOC system. This decrease consequently inhibits the nucleation and growth of Phase 5 crystals and alters their growth morphology. Furthermore, α-MgO reacts with RM to form amorphous gel-like substances. These gel-like substances and the excess RM adhere to the surface and fill the MOC pores to refine the pore structure, thereby increasing the density, blocking the permeation of water molecules, and improving water resistance. Compared with R2C0, the Phase 5 whiskers in R2C1.5 are more slender, exhibit a clustered aggregation pattern, and possess reduced interwhisker spacing. Furthermore, these acicular Phase 5 crystals are bonded by a gelatinous material, forming a dense, network-like structure. This microstructure provides protective encapsulation for the Phase 5 crystals, effectively inhibiting their hydrolysis. This microstructure enables gel-like substances and RM particles to adhere to the surface of Phase 5 crystals, thereby forming a physical isolation layer that reduced the likelihood of direct contact between water molecules and Phase 5 crystals, consequently delaying the hydrolysis process.

The above findings reveal that RM serves as an effective pore-filling agent that stimulates the formation of cementitious gels in RM-MOC, while also inhibiting the formation and development of Phase 5 crystals. By contrast, C_6_H_8_O_7_ not only facilitates the formation and refines the morphology of Phase 5 whiskers, it also contributes to gel formation, leading to a denser microstructure.

**Fig. 14:**
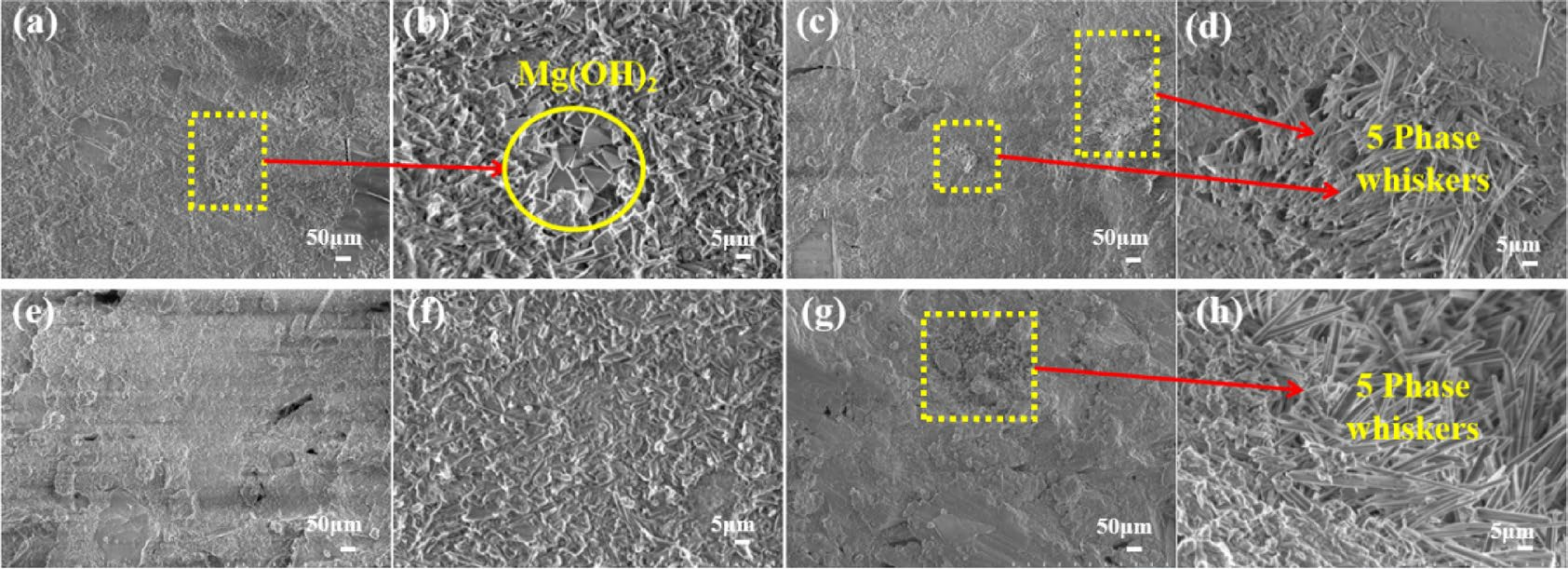
SEM images of MOC samples at different magnifications after immersion in water for 28 d: (**a**) R2C0 at 50 μm; (**b**) R2C0 at 5 μm; (**c**) R2C1.5 at 50 μm; (**d**) R2C1.5 at 5 μm; (**e**) R6C0 at 50 μm; (**f**) R6C0 at 5 μm; (**g**) R6C1.5 at 50 μm; and (**h**) R6C1.5 at 5 μm.

Following immersion in water for 28 d, the Phase 5 crystals in RM-MOC decomposed, generating expansive brucite, which typically exhibits a flaky morphology. As shown in Fig. 14b, R2C0 contains numerous such crystals. Specifically, the SEM image of R2C0 soaked in water shows that almost all rod-like crystals disappeared, forming block- and flake-like Mg(OH)_2_, which exhibits significantly lower strength than needle-like Phase 5. In contrast, needle-like Phase 5 was not observed in R6C0, although the surface consisted of a denser cementing material, thereby accounting for the higher water resistance of R6C0 compared with R2C0. Meanwhile, Fig. 14b and f show that R2C1.5 and R6C1.5 retain substantially elongated Phase 5 whiskers. Overall, the water-blocking effect of the cementitious materials protect Phase 5 crystals from hydrolysis and enhance the water resistance of RM-MOC.

#### SEM-EDS and mapping analysis

**Fig. 15:**
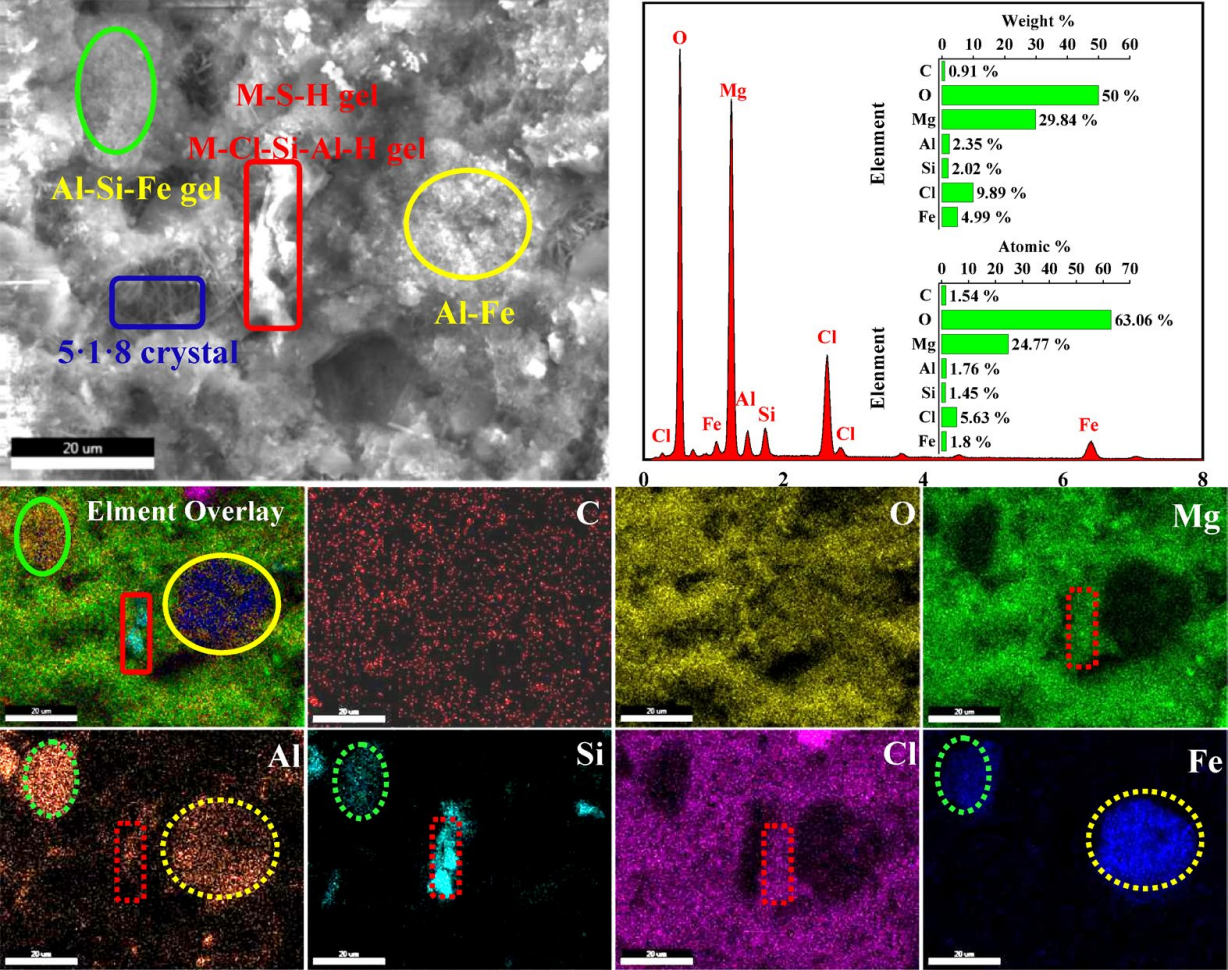
SEM-EDS and elemental mapping analyses of R6C1.5 after curing for 28 d.

To analyze the effects of RM/C_6_H_8_O_7_ on the hydration products of MOC, SEM-EDS and elemental mapping analyses were performed on R6C1.5 after curing for 28 d (Fig. 15). In addition to the needle/rod-like Phase 5 crystals, abundant flocculent and gelatinous substances containing Mg, Cl, and O can also be observed on the R6C1.5 surface. The Mg: Cl: O amount-of-substance ratios were 4.9:1:11.1, indicating compositions similar to the gel-like Phase 5 or Mg(OH)_2_. Furthermore, certain amounts of C, Al, Fe, and Si were detected. Moreover, it was observed that the elemental distribution on the R6C1.5 surface was not uniform and contained areas of Mg, Cl, Si, and Al enrichment. Combined with the TG-DTG results, the gel materials were inferred to be M-S-H gel and Mg-Cl-Si-Al-H gel. Zones enriched with Fe, Si, Al, and Fe were also detected, which exhibited a dense microstructure. This can be attributed to the stable crystalline phases in the RM, including Fe_2_O_3_, SiO_2_, and Al_2_O_3_, which did not participate in the reaction. These phases persist in the matrix as pristine particles, enhancing the densification and mechanical strength of the matrix to produce a heterogeneous structure.

## Discussion

This study systematically investigated the synergistic mechanisms of RM and C_6_H_8_O_7_ in the RM-MOC system. The results demonstrate that, despite their distinct roles, the combined incorporation of both components produces a pronounced synergistic effect, enabling the simultaneous improvement of workability, mechanical strength, and durability in the resulting RM-MOC composites.

### Mechanism of microstructural evolution

The SEM images shown in Fig. 13e and h reveal that specimens with an elevated RM content (i.e., R6C0 and R6C1.5) exhibit finer pores and a more compact microstructure, corroborating the pore size distribution data. This was mainly attributed to fine RM particles acting as micro-aggregates that fill the interstices of the Phase 5 crystal network, subdividing large pores into smaller ones, while simultaneously lowering the effective concentration of α-MgO, which suppresses crystal growth and reduces the volume of large pores, ultimately refining the pore structure. Upon the addition of 1.5% C_6_H_8_O_7_, the Phase 5 whiskers became more slender, aggregated into clusters, and exhibited gel-covered surfaces. Compared with R2C0, the harmless pore content of R2C1.5 increased from 38.62% to 64.25%, while the harmful pore content was reduced from 19.50% to 6.29%. This can be attributed to the fact that the chelating effect of C_6_H_8_O_7_ controls Mg^2+^ release, retards hydration, and promotes a fine, uniform needle-like crystal network that refines the pore structure. Furthermore, the reactive Al_2_O_3_ and SiO_2_ components, originating from RM, dissolve during the retarded hydration period and subsequently react with Mg^2+^ and Cl^−^ to yield amorphous gels (i.e., M-S-H and Mg-Cl-Si-Al-H). These gels infiltrate the microcracks and transform residual harmful pores into harmless or closed pores.

Following 28 d of water immersion, the Phase 5 crystals in R2C0 were almost completely hydrolyzed into flaky Mg(OH)_2_ (Fig. 14b). In contrast, R2C1.5 and R6C1.5 retained abundant slender Phase 5 whiskers, with their surfaces encapsulated by a gel. This indicates that the dense crystal network and gel encapsulation layer induced by C_6_H_8_O_7_ not only refine the pore structure but also block water ingress, ultimately protecting Phase 5 from hydrolysis and maintaining pore integrity. Overall, RM and C_6_H_8_O_7_ demonstrate a pronounced synergistic effect in optimizing the pore microstructure of MOC.

### Retardation mechanism

Increasing the dosage of both RM and C_6_H_8_O_7_ resulted in a prolonged setting time for MOC (Fig. 5), which can be attributed to distinct mechanisms. Specifically, the retarding effect of RM primarily stems from a physical dilution effect, wherein the inert RM particles reduce the concentration of reactive MgO and Cl^−^ species per unit volume, thereby delaying the nucleation and growth rate of Phase 5^48^. Additionally, the adsorption of dissolved species in the solution by the high-specific-surface-area RM particles may further hinder the nucleation process. By contrast, the introduction of C_6_H_8_O_7_ provides a chemical regulation mechanism. For instance, the citric acid carboxyl groups exhibit a strong chelating ability toward Mg^2+^, forming unstable magnesium citrate complexes that effectively control the concentration of free Mg^2+^ in solution^49^. This “chelation-controlled release” mechanism significantly inhibits the rapid precipitation of the initial hydration products, creating favorable conditions for the subsequent orderly and sufficient growth of Phase 5 crystals. When both are incorporated together, the retardation mechanism can be attributed to the superposition and synergy of physical and chemical effects. The fine particle size and adsorptive properties of RM impede the hydration and crystallization processes, whereas C_6_H_8_O_7_, a known chelating agent, complexes with magnesium ions in the solution. This interaction inhibits the nucleation and growth of strength-providing phases, such as Phase 5, thereby further delaying setting. From a practical perspective, this synergistic retardation significantly enhances workability by extending the usable time of the paste and moderating hydration heat evolution.

### Mechanism underlying the maintained compressive strength

The continuous decline in RM-MOC strength, which is induced by RM, is fundamentally attributable to the volume and dilution effects. Although its micro-aggregate effect and weak pozzolanic activity^15^ contribute partially to matrix densification^50^, they are insufficient to compensate for the strength reduction resulting from the loss of the principal crystalline phase. The incorporation of C_6_H_8_O_7_ leads to lower early strength due to its retarding effect; however, it significantly enhances the long-term strength. The authors consider that the synergistic improvement in the mechanical performance of MOC upon RM and C_6_H_8_O_7_ incorporation manifests primarily in two aspects. First, the complexation effect of C_6_H_8_O_7_ slows the MgO hydration rate, promoting the formation of a denser and more stable Phase 5 crystal network, as evidenced by the SEM micrograph of R2C1.5 (Fig. 13). The XRD and FTIR results confirmed that C_6_H_8_O_7_ facilitates the formation of Phase 5 (3610 cm^− 1^) while suppressing the formation of Mg(OH)_2_ (3692 cm^− 1^). Second, the retardation effect of C_6_H_8_O_7_ provided sufficient time for the dissolution of reactive Al_2_O_3_ and SiO_2_ species from the RM, yielding SiO_4_^4−^ and AlO_2_^3−^ species^51^. These dissolved ions subsequently react with Mg^2+^ and Cl^−^ in the system, leading to the formation of amorphous M-S-H gel or Mg-Cl-Si-Al-H gels, which can be observed in the elemental mapping images as regions enriched with Mg, Si, Cl, and Al. These gel phases refine the pore structure, densify the microstructure, and considerably enhance both the mechanical performance and water resistance of the matrix^23,52^. Consequently, even with 60% RM incorporation, the RM-MOC specimens achieved a compressive strength of 59 MPa, exceeding ordinary Portland cement. This demonstrates that the composite retains satisfactory mechanical properties and complies with the strength requirements for construction materials. Furthermore, these findings provide a theoretical basis for the high-volume utilization of RM in MOC production, while simultaneously reducing the production cost of MOC.

### Mechanism for the enhanced water resistance

Pore structure analysis indicates that the hybrid incorporation system achieved a refined reconstruction of the sample porosity, as characterized by a sharp decrease in the proportion of harmful pores and a significant increase in the volume of harmless pores (e.g., the harmless pore content in R6C1.5 increased to 69.38%). Liu et al^53^. found that the reduction in porosity (from 3.35% to 1.65%) and the increase in the proportion of harmless pores are key microstructural reasons for the improvement in material toughness and durability in their study on metakaolin-modified ultra-high performance concrete. This microstructure considerably reduces pore connectivity and effectively hinders the capillary transport of moisture^54^. The high-volume RM particles act as micro-aggregates, directly filling and segmenting pores to provide a robust physical barrier^28,55^. Meanwhile, C_6_H_8_O_7_ promotes the formation of more stable and hydrolysis-resistant Phase 5 crystals^56^. The generated hydration gel phases (M-S-H and Mg-Cl-Si-Al-H gels) exhibit higher chemical stability and possess stronger resistance to moisture attack. Most critically, the SEM morphology of the water-immersed samples (Fig. 14) directly confirms the “encapsulation and protection” mechanism: after immersion, a large number of Phase 5 crystals in the hybrid system were encapsulated and protected by a dense composite structure formed by the cementitious gels and RM particles, isolating them from direct water contact and fundamentally inhibiting hydrolysis. Hence, the synergistic effect of RM and C_6_H_8_O_7_ establishes a multi-scale barrier structure within the MOC matrix, significantly enhancing its water resistance.

In summary, RM and C_6_H_8_O_7_ establish a strong synergistic enhancement effect within the MOC system. C_6_H_8_O_7_ acts as a chemical regulator, controlling the hydration kinetics via chelation and optimizing the structure of the principal crystalline phase. RM serves as both a physical filler and a reactant: it provides a micro-aggregate filling effect to enhance the pore structure, while also supplying elements such as Si and Al for participation in secondary hydration reactions and incorporation into the new gel phases. Through this synergistic interaction, a highly dense composite system is constructed within the MOC matrix. This system features a stable framework of Phase 5 crystals, a dense and multi-phase gel matrix, and reinforcement from RM particles. The resulting structure exhibits outstanding mechanical properties and superior water resistance. However, the strong alkalinity and potential heavy metal toxicity of RM must be considered when evaluating the environmental safety of RM-MOC. Leaching toxicity tests, pH measurements, heavy metal immobilization assessment, and life cycle analysis should therefore be performed in future studies.

## Conclusions

This study investigated the mechanisms responsible for the synergistic effect of RM and C_6_H_8_O_7_ on the workability, water resistance, and mechanical properties of RM-MOC. The main findings are summarized as follows:

(1) RM and C_6_H_8_O_7_ were found to prolong the setting time of MOC. The incorporation of RM alone reduced the compressive strength of the MOC samples, whereas adding C_6_H_8_O_7_ slowed the decrease in compressive strength. Upon the addition of 60% RM, the compressive strengths of R6C1 and R6C1.5 reached 59 MPa, reflecting excellent mechanical properties.

(2) The softening coefficient of RM-MOC increased with increasing RM content, and the incorporation of C_6_H_8_O_7_ led to enhanced water resistance. The RM-MOC sample containing 60% RM and 1.5% C_6_H_8_O_7_ exhibited softening coefficients of 0.80 and 0.78 after 7 and 28 d of water immersion, respectively, demonstrating optimal water resistance.

(3) Under the synergistic effect of RM and C_6_H_8_O_7_, M-S-H and Mg-Cl-Si-Al-H gels possessing a homogeneous and highly polymerized structure were formed in the MOC. These insoluble and dense gel phases refine the pore structure of RM-MOC, protect the Phase 5 crystals from hydrolysis, and consequently enhance both compressive strength and water resistance.

(4) This study achieved high-volume utilization of RM in MOC while avoiding the introduction of high-cost admixtures, providing a new strategy for developing high-performance, low-cost, and low-carbon magnesium-based cementitious materials. Additionally, the effect of RM addition on the workability of RM-MOC was not examined herein and should be a focus of future work.

## Supplementary Information

Below is the link to the electronic supplementary material.

Supplementary Material 1

## Data Availability

The data that support the findings of this study are available from the corresponding author upon reasonable request.
